# Hidden Contamination Patterns: A Stochastic Approach to Assessing Unsymmetrical Dimethylhydrazine Transformation Products in Kazakhstan’s Rocket Crash Area

**DOI:** 10.3390/toxics13110963

**Published:** 2025-11-06

**Authors:** Ivan Radelyuk, Aray Zhakupbekova, Alua Zhumadildinova, Artem Kashtanov, Nassiba Baimatova

**Affiliations:** 1 Department of Chemistry and Chemical Technologies, Toraighyrov University, Pavlodar 140008, Kazakhstan; 2 Faculty of Chemistry and Chemical Technology, Center of Physical Chemical Methods of Research and Analysis, Al-Farabi Kazakh National University, Almaty 050040, Kazakhstan; 3 Environmental and Analytical Chemistry Laboratory, Al-Farabi Kazakh National University, Almaty 050040, Kazakhstan

**Keywords:** soil analysis, transformation products, unsymmetrical dimethylhydrazine, rocket fuel, Kazakhstan, contamination transport modeling, Vac-HS-SPME-GC-MS

## Abstract

Unsymmetrical dimethylhydrazine (UDMH), a highly toxic rocket propellant, remains a significant environmental concern in Kazakhstan due to repeated rocket stage falls near the Baikonur Cosmodrome. This study integrates chemical analysis with stochastic contamination transport modeling to evaluate the persistence and migration of UDMH transformation products (TPs) in soils collected 15 years after the rocket crash. Vacuum-assisted headspace solid-phase microextraction coupled with gas chromatography–mass spectrometry (Vac-HS-SPME-GC-MS) was used to determine five major TPs. Among these, pyrazine (PAN) and 1-methyl-1H-pyrazole (MPA) were consistently detected at concentrations ranging from 0.04–2.35 ng g^−1^ and 0.06–3.48 ng g^−1^, respectively. Stochastic simulations performed with HYDRUS-1D indicated that the long-term persistence of these compounds is mainly controlled by physical nonequilibrium transport processes, including diffusion-limited exchange, weak sorption, and slow inter-domain mass transfer, rather than by degradation. Sensitivity analysis demonstrated that low dispersivity and diffusion coefficients enhance solute retention within immobile domains, maintaining residual levels over extended periods. The results demonstrate the efficacy of combined long-term monitoring and predictive modeling frameworks for assessing contamination dynamics in rocket impact zones.

## 1. Introduction

**Figure 1:**
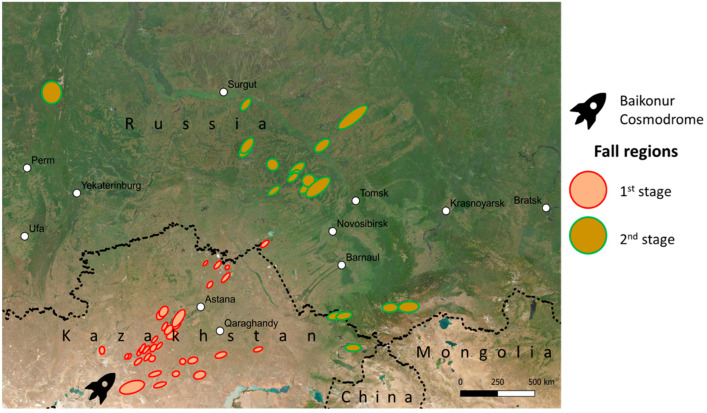
Falling zones of the rocket stages launched from the Baikonur Cosmodrome.

Unsymmetrical dimethylhydrazine (1,1-dimethylhydrazine, UDMH) remains a critical rocket fuel propellant globally due to its specific properties, including high thermal stability, storability, and low freezing point [1]. Despite its high toxicity and environmental persistence [2], UDMH is widely used in Russia, China, and India [3], with Russia being the largest consumer, primarily through rockets such as the Proton and Soyuz [2,4]. The Baikonur Cosmodrome, established in 1957 in Kazakhstan (leased by Russia), stands as the world’s first, oldest, and largest [5] operational space launch facility, responsible for over 70% of Soviet/Russian launches [2,5]. The Baikonur Cosmodrome conducted 10 rocket launches fueled by UDMH annually, which consistently release the fuel to the environment during the flight and separation stages [6]. Rocket launches are accompanied by the fall of separated parts (rocket stages) to the ground (Figure 1) [7,8,9]. Between 1999 and 2018, six accidents involving rocket launches occurred, resulting in a severe environmental impact. A crash of the Proton M rocket in September 2007 led to a massive fire erupting at the fall site and leakage of 219 tons of UDMH with NO_2_ as an oxidant. The crash landing of the Proton-M rocket occurred at the cosmodrome, approximately 2.5 km from the launch site, resulting in a spill of 600 tons of fuel in July 2013. According to the United Nations Development Program [10], the affected fall zones, comprising cropland, pastures, and other areas, covering an area of 77.09 million hectares, have been classified as disaster zones.

Studies have shown that UDMH can persist in soil for 90 days; however, after this period, its concentration drops below 0.5% of the initial amount [11]. Therefore, transformation products (TPs) of the UDMH are detectable in soil even 30 years after discharge [12,13]. To date, a total of 88 transformation products of UDMH have been detected and confirmed [1]. Part of the transformation products are mostly intermediate products of the oxidation, degradation, or other reactions of the UDMH. The 1-formyl-2,2-dimethylhydrazine (FDMH) is characterized as an unsaturated compound, which easily hydrolyzes and oxidizes to N-nitrosodimethylamine (NDMA) and dimethylamine (DMA). N-nitrosodimethylamine (NDMA) in particular is recognized as more carcinogenic and highly toxic compared to UDMH [14]. Another major transformation product, 1-methyl-1H-1,2,4-triazole (MTA), is known for its highest concentrations in soils and is the most stable among all TPs [15]. The carcinogenic, mutagenic, and persistent nature of UDMH and its residual compounds raises serious concerns about their impact on both the environment and human health [16,17,18]. Their continuous monitoring is important due to their carcinogenic, toxic, and stable characteristics. However, volatility, reactivity, and diversity of the UDMH TPs pose challenges in preparing methods for their determination. Despite growing interest in the environmental and toxicological impacts of UDMH and its transformation products, there is limited information available about their migration, redistribution in different soil layers, and remediation in contaminated areas [19]. Due to the detection of UDMH TPs even after decades of pollution, their screening and continuous monitoring are essential for future remediation and development of remediation strategies.

Studies on real samples from the Baikonur region demonstrated results on water [20], snow [21], and soil samples. Soil samples were analyzed using high-resolution Orbitrap mass spectrometry with sample preparation via pressurized liquid extraction (PLE), although this method requires a large amount of solvents [22]. Kenessov et al. [12] applied gas chromatography-mass spectrometry (GC-MS) with sample preparation techniques as solvent extraction (SE) and solid-phase microextraction (SPME). In comparison to SE, the results of SPME-based analysis showed promising results for volatile TPs, providing higher sensitivity and cost-effectiveness. Analysis with thermal desorption coupled to GC-MS identified 62 UDMH TPs, including new compounds [23]. Similarly, Bakaikina et al. [24] analyzed samples using GC-MS/MS with SPME and accelerated solvent extraction (ASE), resulting in SPME with lower detection limits for analytes with boiling points < 180 °C compared to ASE. Recently, Kurmanbayeva et al. [25] applied a gas chromatography- nitrogen-phosphorus detector (GC-NPD) for on-site analysis of UDMH TPs using vacuum-assisted headspace SPME (Vac-HS-SPME).

Among existing analytical techniques, headspace-SPME (HS-SPME) coupled with GC-MS exhibits high sensitivity and selectivity, along with environmental sustainability [24,26,27,28]. However, the use of the HS-SPME is limited by matrix effect and extraction kinetics. To address these challenges, Brunton et al. [29] optimized the pressure settings, resulting in a 5-fold increase in responses. Vac-HS-SPME showed an increase in responses and a reduction in extraction time [30,31,32]. The study on the determination of UDMH TPs using Vac-HS-SPME-GC-MS demonstrated increased sensitivity, along with improved accuracy and precision [32].

Despite advances and acknowledgments of the environmental impact of UDMH transformation products, limited studies have explored the hidden contamination patterns in soil at vertical levels and the migration patterns of these contaminants. The homogeneity of the impacted zones limits standard assessment methods, whereas integrating stochastic methods may provide a holistic overview of long-term fate assessment and projected regulatory challenges. The HYDRUS software is frequently used as a computational platform for stochastic and uncertainty analyses in soil systems, as it employs a numerical framework to simulate water flow and solute transport in variably saturated porous media [33]. Several studies have demonstrated the feasibility of stochastic simulations with HYDRUS in unsaturated soils. Moghbel et al. [34] and Zhou et al. [35] coupled HYDRUS-1D with Bayesian Markov Chain Monte Carlo (MCMC) calibration and Monte Carlo sampling to quantify the posterior uncertainty of hydraulic, sorption, and degradation parameters in soil systems. Their results showed that probabilistic calibration enhances model realism and captures the variability observed in experimental leaching data. Skaggs et al. [36] applied a similar stochastic approach in lysimeter-scale simulations, where random sampling of key soil hydraulic parameters (*θr*, *θs*, *α*, *n*, *Ks*) yielded wide prediction intervals in solute fluxes and demonstrated the sensitivity of transport predictions to parameter uncertainty. Bauser et al. [37] further emphasized that representative parameterization in heterogeneous soils is often unattainable without stochastic treatment, since local variations in hydraulic properties strongly affect content dynamics. These studies demonstrate that stochastic or probabilistic interpretations of soil hydraulic and transport parameters are crucial for reproducing the observed variability in contaminant mobility within the vadose zone.

The aim of this study was to evaluate the behavior of UDMH TPs in contaminated soils. The objectives of this study were a) to assess the depth and the spatial distribution of the UDMH TPs, such as pyrazine (PAN), 1-Methyl-1H-pyrazole (MPA), 1-Methyl-1H-1,2,4-triazole (MTA), and 1H-Pyrazole (PAL), based on results obtained with Vac-HS-SPME-GC-MS analysis; and b) to investigate the potential long-term contamination patterns of UDMH TPs using stochastic contamination transport modeling in soils at the Baikonur Cosmodrome’s areas impacted by launch vehicles.

## 2. Materials and Methods

The methodological framework of this study consists of three main steps: sampling, measurement, and contaminant transport modeling. Step 1 involved collecting samples at the restricted-access crash site of the “Proton-M” launch, with representatives from the Republican State Enterprise “Infrakos”. Step 2 focused on measuring the soil’s pH, moisture content, and organic matter, which are key indicators of soil physicochemical properties affecting contaminant mobility and retention. It also included identifying trace levels of UDMH TPs using Vac-HS-SPME-GC-MS. Finally, Step 3 involved stochastic transport modeling of contamination using the HYDRUS-1D simulation environment.

### 2.1. Study Area

This study investigates the crash site of the “Proton-M” launch in Ulytau district, Karaganda region, located 40 km southwest of Zhezkazgan, Kazakhstan. The incident on 6 September 2007, at Baikonur Cosmodrome involved an unsuccessful launch, resulting in a 45 m by 20 m crater created by the main rocket assembly components, as well as 119 impact sites caused by the fragmentation of the “Proton-M” rocket. At the time of the incident, the carrier rocket still contained more than 218 tons of UDMH propellant. The Russian Federation subsequently confirmed remediation activities at contaminated sites, including areas where UDMH concentrations in soil exceeded Kazakhstan’s regulatory thresholds (0.1 mg/kg) (Table S1) [38].

### 2.2. Soil Sampling Design and Procedure

**Figure 2:**
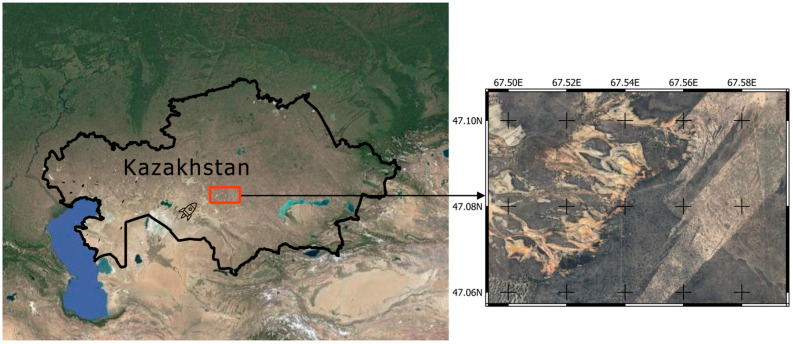
Approximate location of soil sampling area at the crash site of the “Proton-M” launch in Kazakhstan.

Soil sampling was conducted in September 2022, representing a 15-year post-impact timeframe following the crash-landing. The approximate area of the jet fuel spillage was identified between 67°50′ E and 67°57′ E, and 47°06′ N and 47°09′ N (Figure 2). Sampling was accompanied by authorized representatives responsible for remediation, and the exact location was not disclosed due to existing restrictions.

**Figure 3:**
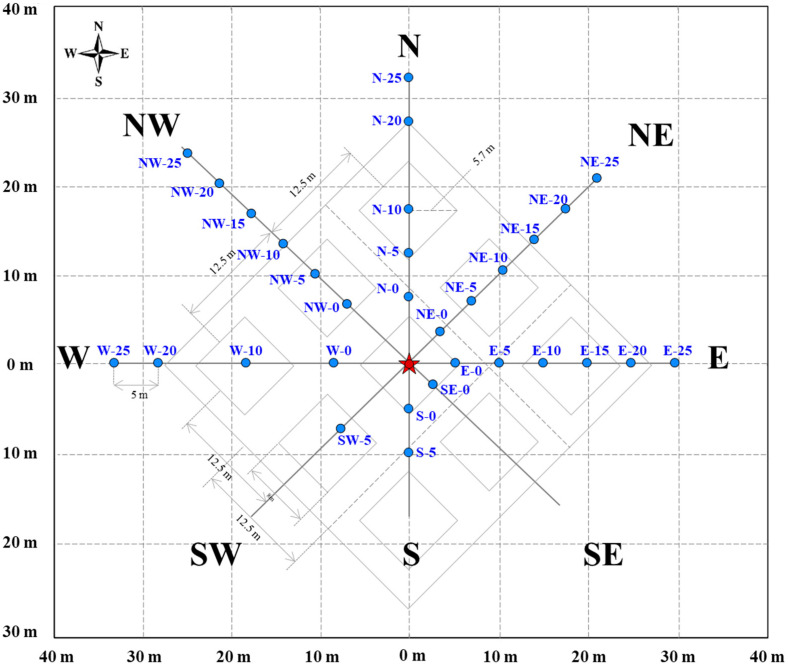
Scheme of the sampling grid map: blue dots indicate sampling points, capital letters denote directions, and the numeric label at each dot represents the radial distance (m) from the epicenter.

Samples were collected using soil corers from the contaminated area along eight radial directions extending from the impact epicenter. The spatial configuration of the sampling grid is illustrated in Figure 3.

The sampling was implemented with the orientation directed from the crash epicenter toward the most affected part of the study area. From this central point, eight lines were laid out along the cardinal and intercardinal directions (N, NE, E, SE, S, SW, W, NW). On the perimeter of the central zone, seven sampling points were established and designated as zero-distance reference locations. Along each transect, additional sampling points were positioned at 5-m intervals. At each point, a borehole was drilled using a specialized corer. Depth-specific samples were collected at 50 cm, 100 cm, and 150 cm at each location. Borehole depth was measured and recorded prior to coring at the target intervals. Three replicate cores were obtained at each depth to assess local variability and support statistical analysis. The total sampling area covered approximately 2500 m^2^, with 5-m spacing between consecutive locations along each transect. A total of 96 soil samples were collected.

### 2.3. Physical and Chemical Characteristics of Soils

Characterizing soil physical and chemical properties enables the effective grouping of field samples based on matrix composition, facilitates reliable calibration, and enhances the accuracy and comparability of UDMH TPs quantification. Matrix effects in soils are primarily influenced by moisture content, organic matter, and pH. To minimize these effects, soils with similar characteristics were grouped, and GC-MS calibration was performed separately for each matrix group.

Moisture content, organic matter, and pH were determined according to standardized methods (GOST 28268-89 [39], GOST 27784-88 [40], and GOST 26423-85 [41], respectively). Each parameter was measured once as part of the initial matrix characterization, which is sufficient for the intended purpose of classifying samples into comparable matrix categories.

Moisture content was determined using 5.0 ± 0.1 g of soil weighed on ALC 210.4 (ACCULAB Sartorius Group, Göttingen, Germany) balances and conditioned in an SNOL 8.2/1100 (SNOL, Utena, Lithuania) oven at 105 °C for 4–6 h, until the mass difference was <0.1 g (Table S2). The moisture content varied from 1.4% to 12.9% for the samples.

Organic content was quantified by soil (5.0 ± 0.1 g) loss-on-ignition at 105 °C for 4–6 h, then combusted at 525 °C for 5–7 h until mass change was <0.001 g. The measured organic matter content of the soil samples ranged from 1.3% to 8.2% (Table S2).

Soil acidity (pH) was measured from a soil–water extract (1:5 ratio, 10.0 ± 0.1 g soil to 50 mL of deionized water (DIW)). The suspension was agitated for 5 min using a magnetic stirrer (Stegler HS, Ningbo, China), followed by 5 min of sedimentation. The supernatant pH was measured with a calibrated pH meter (HANNA Instruments, Smithfield, RI, USA) over the pH range of 4.0 to 10.0. Soil pH consists of 7.0–10.1 (Table S2).

### 2.4. Sample Preparation

The sample preparation followed a previous study using Vac-HS-SPME [29]. A 1.0 ± 0.1 g soil sample, sieved through a 3-mm mesh, was weighed into a 20 mL glass vial. The vial was air-evacuated for 20 s using a rotary vane pump at 0.3 Pa (Stegler JK-2VP-2, Ningbo, China). Extraction was performed using an 85-µm Carboxen/polydimethylsiloxane (Car/PDMS, Supelco, Bellefonte, PA, USA) fiber at a temperature of 40 °C for 30 min during both incubation and extraction [32]. The analysis was carried out in triplicate at each sampling site, with depths of 50 cm, 100 cm, and 150 cm.

### 2.5. Calibration and Analytical Performance

UDMH TPs, such as PAN (99%, Meryer (Shanghai) Chemical Technology Co., Ltd., Shanghai, China), MPA (98%, Meryer (Shanghai) Chemical Technology Co., Ltd., China), MTA (98%, Meryer (Shanghai) Chemical Technology Co., Ltd., China), PAL (99%, Meryer (Shanghai) Chemical Technology Co., Ltd., China), and NDMA (99%, Shanghai Macklin Biochemical Co., Ltd., Shanghai, China), were used to prepare standard solutions in deionized water (DIW) produced by the YoungLin Ultra series 370 water purification system (Young Lin Instrument Co., Ltd., Anyang, Republic of Korea). Stock solutions of the analytes were prepared in DIW, stored in 20 mL amber glass vials (Zhejiang Aijiren Technology Inc., Quzhou, China), and kept frozen at −20 °C until further analysis.

MS detector calibration employed a group-specific approach with seven distinct soil matrices (3 for a 50 cm depth, 2 for a 100 cm depth, and 2 for a 150 cm depth) containing negligible analyte levels (<0.1 ng g^−1^). Groups were defined by similar physicochemical profiles (pH, moisture, organic content) identified through PCA analysis (Table 1): SE 0–50; E 5–50; E 10–50; E 20–100; C 100; E 5–150; N 20–150.

The standard addition method was applied to account for matrix effects, with the following linear calibration ranges: PAN, MPA, and NDMA from 2.6 to 100.0 ng g^−1^, PAL from 13 to 500 ng g^−1^, and MTA from 52 to 2000 ng g^−1^. A soil sample in a 20 mL glass vial was spiked with 10 µL of the standard solution according to the analyte-specific calibration ranges (Table S3), and three replicates of each concentration level for the selected soil matrix were prepared. The spiked soil was vortexed in MS 3 basic (IKA, Baden-Württemberg, Germany), stored for 12 h at 23 °C for equilibration, and then kept in a freezer at –30 °C.

Group SE 0-50 required triplicate calibration due to signal instability. Calibration curves exhibited determination coefficients (*R*^2^) of 0.934–0.999 across all analytes and groups, except PAL in the soil group of E 5-150 (*R*^2^ *=* 0.807) (Table 2). Relative standard deviations (RSDs) of the curve slopes ranged from 1% to 22% for all studied analytes. Limits of detection (LOD) and quantification (LOQ) were determined from signal-to-noise (S/N) ratios of 3:1 and 10:1 at the lowest calibrated concentrations, yielding ranges of 0.16–91.80 ng g^−1^ (LOD) and 0.524–306 ng g^−1^ (LOQ) (Table 2).

### 2.6. GC-MS Analysis of UDMH TPs

All analysis were performed using the Chromatec Crystal 5000.2 GC-MS (Yoshkar-Ola, Russia). The analytes were desorbed from the Car/PDMS SPME fiber in the GC inlet in splitless mode at 250 °C using a 0.75-mm i.d. liner (Chromatec, Yoshkar-Ola, Russia) for 11 min. Separation of UDMH TPs was performed on a polar 30 m × 0.25 mm HP-INNOWax column with a 0.25 µm film thickness (Agilent, Santa Clara, CA, USA) at a constant helium flow rate of 1 mL/min (>99.995%, Orenburg-Techgas, Orenburg, Russia). The oven temperature was programmed to heat from 80 °C (held for 3 min) to 100 °C at a heating rate of 10 °C/min (held for 2 min) and then ramped to 250 °C at 25 °C/min (held for 6 min). The total GC run time was 11 min. The ion source and MS interface temperatures were set to 230 °C and 260 °C, respectively. MS detection was conducted using electron impact ionization at 70 eV in the selected ion monitoring (SIM) mode. The MS program used for UDMH TP detection in SIM mode is provided in Table 3.

### 2.7. Stochastic Assessment Framework

Considering site access limitations that restrict detailed field investigations, highly reliable analytical models for contaminant transport were infeasible. As a result, forecasting solute concentrations in the vadose zone and identifying transport and retention parameters that explain long-term persistence of contamination at monitoring depths presents a challenge. Therefore, stochastic modeling with related sensitivity analysis was conducted on available measurements to identify potential patterns in the behavior of UDMH TPs in the collected soils.

The HYDRUS-1D software (version 5.04) served as the modeling platform, as it is specifically designed for assessing contaminant transport simulation in vadose zones [42]. Dual-porosity models were applied to account for physical and chemical non-equilibrium transport processes, capturing contaminant exchange between mobile and immobile domains, which is a primary cause of long-term tailing [43]. The hypothesized slow mobility and multi-year persistence of TPs in the subsurface justified this approach. Measured soil properties indicated a low water content [44], moderate to high pH levels [45,46], and a low organic matter content [47], supporting the assumption of dominant equilibrium sorption with gradual non-equilibrium transfer to the solid phase. Initial simulations employed equilibrium sorption as the baseline condition.

For water flow boundary conditions, a constant pressure head was applied at the upper boundary, with free drainage at the bottom, typical for deep vadose zone modeling without a fixed water table. Solute transport was simulated using a concentration flux (Cauchy, or third type) boundary condition at the top and a zero-concentration gradient condition at the bottom, allowing free outflow.

The following parameters were selected for the sensitivity analysis: the linear sorption coefficient (*Kd*), immobile water content (*θ*), molecular diffusion coefficient (*Diffus. W*), the longitudinal dispersivity (*α*), the first-order mass-transfer coefficient between mobile and immobile water domains (*α*′), and the first-order kinetic sorption coefficient (ω).

Inverse modeling was performed to estimate baseline conditions, using measured solute concentrations at various depths as the objective function. Minimization of the objective function is achieved through the Levenberg–Marquardt nonlinear minimization method, which combines elements of both Newton and steepest descent methods, and generates confidence intervals for the optimized parameters [48,49]. Using this inverse modeling framework, a subset of transport and retention parameters was chosen for calibration, while others were constrained to direct measurements, representative estimates, or literature-based values. Iterative runs were extended beyond the default range of 10–50 steps as required to ensure convergence, which was assessed by a sustained reduction in the sum of squared residuals and stabilization of parameter updates. Additional checks included verifying numerical mass-balance errors to confirm model stability and inspecting the parameter correlation matrix to evaluate identifiability and possible trade-offs, particularly between *Kd* and *α*. The plausibility of estimated parameters was further validated by comparison with established literature ranges for soils with similar textures and organic matter content.

## 3. Results

### 3.1. Contamination Profile

Among the five studied transformation products, only PAN and MPA were consistently identified at different depths within the crash site area. Neither NDMA, MTA, nor PAL was found in any of the collected samples. Concentrations of PAN and MPA varied between samples from different depths. The soil at 50 cm demonstrated the highest saturation, with both the highest number of kernels containing the pollutants and the highest average concentrations of the pollutants (Table 4). Average concentrations of pyrazine decreased gradually with depth, showing a two-fold reduction at each depth. At 100 cm, pyrazine was identified in 22 kernels, and at 150 cm, in 18 kernels. Loadings of MTA also decreased with depth, showing a three times lower concentration at 150 cm compared to 50 cm. However, it was only found in a single specimen from a depth of 100 cm.

**Figure 4:**
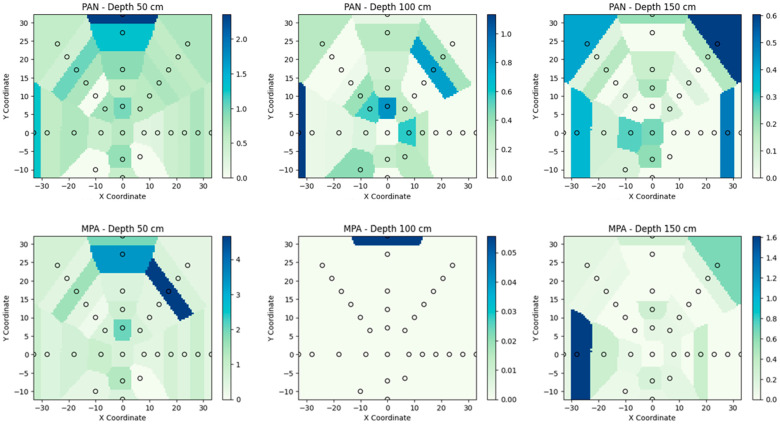
Mapping of concentrations of PAN and MPA in soil samples at depths of 50 cm, 100 cm, and 150 cm.

Lateral distribution maps of the pollutants are illustrated in Figure 4. At a depth of 50 cm, both UDMH TPs tend to spread ununiformly with a few highly concentrated zones. Distribution of PAN and MPA along the cardinal directions was not observed.

However, for PAN, both the skewness and kurtosis of its concentration distribution within a given soil layer decrease with depth, indicating a progressive homogenization of the compound’s concentration in the soil. In contrast, MPA demonstrates an increase in skewness and kurtosis at the 150 cm mark. Since both pollutants are water-soluble, this behavior could suggest a tendency for MPA to accumulate through specific chemical interactions with soil. For example, the CH_3_ group of MPA provides hydrophobicity to the molecule, which may lead to favorable interactions with organic compounds [3,50]. The organic content in the soil samples associated with the peak MPA concentrations was average compared to other samples from the same depth.

**Figure 5:**
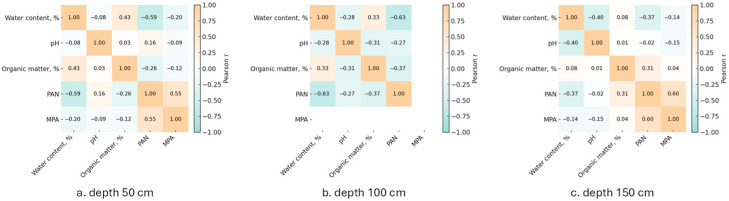
Heatmaps of pairwise Pearson correlations between soil physicochemical parameters and concentrations of PAN and MPA by depth: (**a**) 50 cm; (**b**) 100 cm; (**c**) 150 cm.

The correlation between pollutant concentration and these soil characteristics is visualized in Figure S1. Prominent trends can be observed for the three 50 cm soil groups. Soils with lower water content and higher acidity exhibited a higher average concentration of both PAN and MPA. At the same time, the organic matter content of all three soil groups fell within a range where no consistent relationship was observed between pollutant concentrations and the organic matter content. Pearson correlation analysis supports these findings, showing a moderate to significant negative correlation (r = −0.37 to −0.63) between soil water content and PAN concentration (Figure 5). In the case of more hydrophobic MPA, the correlation was weak (r = −0.14 to −0.20). Correlation with acidity and organic matter content was also not significant (|r| < 0.31). However, the correlation with organic content changed from positive at 50 cm to negative at 100 cm and 150 cm, which could be due to a shift in soil composition.

### 3.2. Stochastic Modeling Results

The inverse modeling yielded calibrated parameters within expected ranges for soils of comparable texture and organic matter content, aligning with existing literature. The optimized parameter remains stable within physically meaningful ranges. The linear sorption coefficient (*Kd* = 14 cm^3^ kg^−1^) corresponded to weak-to-moderate sorption, consistent with reported values for hydrazine derivatives in sandy loam soils with low organic matter content [11,51,52]. This implies retention is dominated by relatively weak mineral matrix interactions rather than strong organic matter partitioning. The fitted mass-transfer coefficient (*α*′ = 4 × 10^−2^ day^−1^) indicated a moderately fast exchange rate between mobile and immobile domains, typical for structured soils characterized by limited immobile water fractions and partial physical nonequilibrium [53]. The longitudinal dispersivity converged to *α* = 0.54 cm, reflecting realistic dispersive spreading at the column scale, in agreement with ranges reported for compounds exhibiting similar aqueous diffusion properties [54,55,56,57]. Point N20 (MPA concentration of 3.36 ppb at a 50 cm depth) was selected as the baseline starting concentration for a 500-day sensitivity simulation.

**Figure 6:**
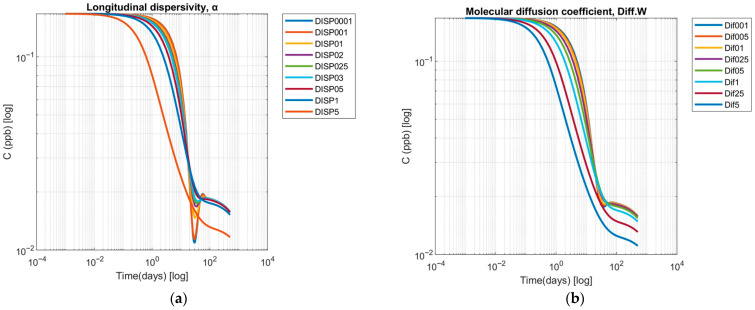
Sensitivity analysis of (**a**) the longitudinal dispersivity, and (**b**) the molecular diffusion coefficient.

Sensitivity analysis for soil hydraulic transport parameters included variations in the longitudinal dispersivity (α) and the molecular diffusion coefficient (*D*). Trends in solute transport with different longitudinal dispersivities (*α* = 0.001–5 cm) illustrate key insights for the long-term fate of UDMH TPs in soils (Figure 6a). During the initial phase, the advection-dominated transport leads to minimal spatial redistribution, suggesting that freshly deposited or recently formed UDMH TPs may remain concentrated near the contamination source. As transport progresses into intermediate timeframes, dispersivity-dependent spreading becomes significant: lower dispersivity values yield dilution, while higher values promote more uniform distribution and accelerated peak attenuation.

The simulation results demonstrate a systematic relationship between solute concentration dynamics and the longitudinal dispersivity. At early times (*t* < 0.01 day), all cases exhibit nearly identical concentrations (0.167 ppb), confirming that advective transport dominates the initial solute distribution, with negligible influence from dispersivity. As time progresses, dispersivity-dependent spreading becomes increasingly pronounced. Low dispersivities (*α* ≤ 0.1 cm) result in modest concentration declines, with minor stabilization at intermediate times, reflecting limited solute spreading and weak redistribution. Intermediate values (*α* = 0.2–0.5 cm) exhibit a more rapid reduction in peak concentration, earlier attenuation, and smoother profiles, indicating enhanced longitudinal mixing. High dispersivities (*α* ≥ 1 cm) demonstrate the fastest local concentration decreases and the broadest, flattest profiles, with late-time asymptotic concentrations reaching minimum values (0.012–0.016 ppb), consistent with efficient solute dispersion over the domain.

These findings suggest that low-dispersion conditions may facilitate the accumulation of UDMH TPs and prolonged contamination risks, whereas higher dispersivity promotes more rapid attenuation of localized maxima but increases the potential for broader soil and groundwater exposure over long timescales. Regardless of dispersivity, the temporal evolution of concentrations shows an initial rapid UDMH TP decay followed by a slow, long-term decline of TPs, highlighting the environmental persistence of these compounds [2].

The analysis of solute concentration dynamics across a range of molecular diffusion coefficients (*D* = 0.01–5 cm^2^ d^−1^) shows distinct diffusion-controlled transport patterns (Figure 6b). At initial timepoints (*t* < 1 day), concentrations remain approximately constant (0.167 ppb) across all *D* values, indicating that initial conditions dominate solute distribution, and diffusive fluxes are negligible.

Over extended periods (1–50 days), concentration patterns divergence: low *D* (0.01–0.1 cm^2^ d^−1^) show minimal concentration reduction (0.016–0.018 ppb at 50 days), reflecting limited solute mobility, whereas medium *D* (0.25–1 cm^2^ d^−1^) and high *D* (2.5–5 cm^2^ d^−1^) lead to progressively larger decreases (0.012–0.013 ppb for *D* = 2.5–5 cm^2^ d^−1^), indicative of enhanced solute spreading and beginning homogenization.

Over extended periods (50–500 days), soils with low *D* values retain higher solute levels (0.015–0.016 ppb), medium *D* soils reach moderate homogenization (0.012–0.014 ppb), and high *D* soils demonstrate substantial flattening of concentration gradients (0.011–0.012 ppb), highlighting diffusion-driven transport. The results identify three distinct transport regimes: diffusion-limited (*D* ≤ 0.1 cm^2^ d^−1^), transitional (*D* = 0.25–1 cm^2^ d^−1^), and diffusion-dominated (*D* ≥ 2.5 cm^2^ d^−1^).

These findings emphasize that early-time predictions are largely insensitive to the diffusion coefficient, whereas long-term solute fate and hotspot persistence strongly depend on *D*. This has important practical implications for risk assessment and remediation strategies, including soil mixing or enhanced bioturbation to promote solute redistribution [58]. By the simulation’s endpoint (*t* = 500 days), the effect of molecular diffusion becomes evident. Systems with higher diffusion coefficients exhibit lower residual concentrations, indicating more effective dispersion of UDMH TPs and potentially slower localized accumulation. Conversely, a low-diffusion scenario maintains slightly higher concentrations, suggesting a higher risk of long-term hotspots in low-diffusivity soils. Thus, the modest differences observed across diffusion scenarios align with the relatively low mobility of UDMH TPs in soils characterized by weak-to-moderate sorption.

**Figure 7:**
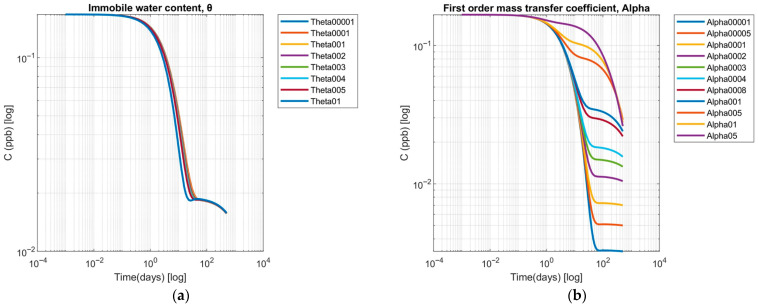
Sensitivity analysis of (**a**) the immobile water content parameter, and (**b**) the first-order mass transfer coefficient.

Sensitivity analysis for soil structural parameters controlling physical nonequilibrium included variations for the first-order mass-transfer coefficient (α′) and the immobile water content (*θ*). Simulations across immobile water content (*θ*) from 0.0001 to 0.1 demonstrated a moderate dependence of UDMH TP persistence on immobile-water fraction (Figure 7a). At the lowest values (*θ* = 0.0001–0.001), the concentration-time profiles were nearly identical to those of single-domain advection-dispersion transport. The mobile-phase concentration remained at 0.167 ppb for the first 0.01 days and then gradually decreased to 0.14 ppb by 1 day and 0.05 ppb by 10 days, without a distinct long-term tail. At *θ* = 0.01–0.02, deviations from advective-dispersive behavior became more noticeable. Concentrations declined more rapidly during the first day (0.12 ppb at 1 day versus 0.14 ppb for *θ* = 0.001) but residual levels of 0.04–0.05 ppb persisted beyond 100 days, indicating solute storage in immobile zones with subsequent gradual remobilization. This effect intensified at *θ* = 0.03–0.05, where mobile phase levels dropped to 0.10 ppb by 1 day and 0.03 ppb by 10 days, yet sustained residual concentrations of 0.02–0.03 ppb for several hundred days. At maximum immobile fraction (*θ* = 0.1), mobile concentrations decreased most rapidly, from 0.167 ppb at 0.001 days to 0.05 ppb at 7 days. However, these concentrations exhibited the highest long-term persistence, maintaining a plateau of 0.018–0.020 ppb over a 500-day simulation period.

These findings demonstrate a non-linear threshold response: *θ* ≤ 0.001 had a negligible effect, *θ* around 0.01 introduced significant long-term tailing, and *θ* ≥ 0.03 produced both accelerated early depletion and enhanced long-term persistence. This behavior reflects diffusion-limited exchange between mobile and immobile regions, consistent with dual-porosity transport theory [42,59].

The sensitivity analysis of the first-order mass transfer coefficient (*α*′) showed significant modulation of UDMH TP persistence in the liquid phase (Figure 7b). This behavior reflects the experimental observation of UDMH and its TPs persisting in natural soils, where immobile pore water fractions serve as long-term contaminant reservoirs [42].

Systematic variation in *α*′ values produced distinct concentration decay kinetics. Low transfer rates (*α*′ ≤ 0.001 d^−1^) resulted in a gradual decline, with concentrations exceeding 0.100 ppb beyond 50 days and remaining above 0.050 ppb after 200 days. Intermediate values (*α*′ = 0.01–0.05 d^−1^) yielded half-lives of 30–60 days, consistent with referenced laboratory biodegradation experiments under aerobic conditions [60]. The highest evaluated rate (*α*′ = 0.5 d^−1^) produced a rapid concentration decline below 0.050 ppb within the first 20 days, comparable to kinetics observed in oxidative transformation pathways such as ozonation and catalyzed degradation [61]. The modeled concentration tailing behavior under low α′ conditions aligns with field reports of residual UDMH and its nitrogenous TPs persisting at measurable levels in soils years after releases of rocket propellant [19,62].

**Figure 8:**
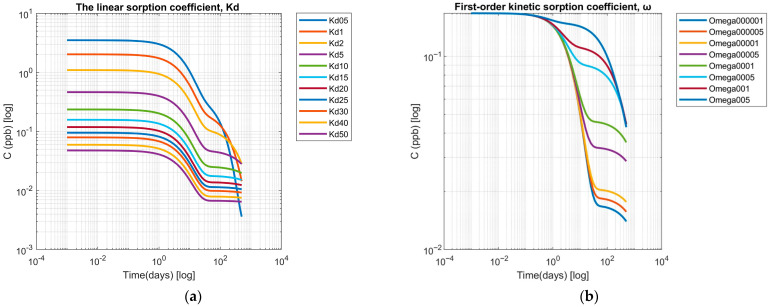
Sensitivity analysis of (**a**) the linear sorption coefficient, and (**b**) the first-order kinetic sorption coefficient.

Finally, sensitivity analysis for soil chemical interaction parameters included variations for the linear sorption coefficient (*Kd*) and the first-order kinetic sorption coefficient (ω). The time-concentration profiles simulated for a solute under *Kd* ranging from 0.5 to 50 cm^3^ kg^−1^ reveal distinct sorption-dependent attenuation patterns (Figure 8a). During the earliest phase (10^−3^–10^−2^ days), all scenarios show nearly identical concentrations (~3.5 ppb for *Kd* = 0.5 cm^3^ kg^−1^) with minimal decline as *Kd* increases, indicating that sorption processes remain negligible when advection-dispersion dominates transport. A noticeable separation between concentration curves emerges between 0.1 and 1 day, reflecting progressively stronger retardation with increasing *Kd*. For intermediate adsorption coefficients (5–15 cm^3^ kg^−1^), attenuation is more pronounced, and concentrations fall below 0.1 ppb within 30 to 60 days, a behavior representative of sandy and loamy soils with low-to-moderate organic carbon content. At high sorption strengths (*Kd* ≥ 20 cm^3^ kg^−1^), concentrations exhibit strong initial retardation and eventually stabilize around 0.01 ppb by 200–250 days, reflecting slower but more persistent release compared to intermediate *Kd* values (5–15 cm^3^ kg^−1^), which decline below 0.1 ppb within 30–60 days. Such behavior suggests strong immobilization typical of soils enriched in clay minerals or organic matter.

Across scenarios, quasi-equilibrium plateaus are approached within 200–250 days, with levels differing by more than an order of magnitude. For *Kd* = 0.5 cm^3^ kg^−1^, concentrations remain above 0.04 ppb, while for *Kd* ≥ 20 cm^3^ kg^−1^ they stabilize near 0.01 ppb. This pattern suggests that long-term tailing is predominantly adsorption-controlled, consistent with dual-porosity transport models, in which slow release from immobile domains sustains low-level concentrations over time.

Variation in the first-order kinetic sorption coefficient (ω) primarily affected the long-term behavior of UDMH TPs in the aqueous phase (Figure 8b). During initial transport stages (*t* < 0.01 d), all simulations yielded identical concentrations (0.167 ppb), indicating negligible sorption kinetics effects on the advective-dispersive front. Significant divergence emerged only after several days of transport, with ω controlling the rate of concentration decline.

Very low values (ω = 10^−5^–10^−4^ d^−1^) resulted in gradual concentration decreases, maintaining levels above 0.100 ppb for more than 100 days and sustaining >0.050 ppb through 300 days, with pronounced tailing. Intermediate rates (ω = 10^−3^–10^−2^ d^−1^) accelerated solute depletion, reaching concentrations of 0.050–0.070 ppb within 100 days. The strongest effect occurred at ω = 0.05 d^−1^, where concentrations declined below 0.050 ppb by day 100, representing about a 40% reduction relative to the slowest kinetic scenario.

These patterns indicate that ω predominantly governs nonequilibrium sorption and the efficiency of solute exchange between mobile and sorbed phases. Low ω values indicate kinetically limited sorption, which enhances solute mobility and persistence, while high ω values approach near-equilibrium exchange, promoting stronger retardation. The persistence of residual concentrations under slow kinetic conditions is consistent with reported field observations of UDMH derivatives in subsurface soils years after initial contamination events [19,62].

## 4. Discussion

Although several methods for determining and quantifying UDMH TPs have been established, consistent long-term monitoring of contaminated sites remains scarce. Due to UDMH TPs persisting post-remediation, evaluating their migration and distribution patterns is essential for developing the most efficient remediation strategies.

The Convention on International Liability for Damage Caused by Space Objects claims that countries are responsible for preventing, mitigating, and remedying environmental contamination from their space activities, and must provide reparation for such damage [63]. Strengthening remediation strategies at rocket impact sites involves detailed investigations of soil hydraulic properties and modeling of contaminant behavior. Studies have shown that current practices are often ineffective, as heptyl and nitrous compounds remain in soil and plants years after detoxification [1,64]. This persistence is largely caused by the oxidative transformations of the primary contaminants, generating hundreds of stable transformation products.

Our research, which combines field observations with stochastic modeling, confirms that accurate risk assessment requires both contaminant measurements and analysis of soil characteristics. This aligns with experimental studies [65,66], UDMH mobility depends on soil acid–base properties, organic matter content, and clay fraction mineralogy. At Baikonur Cosmodrome, impermeable clays and loams restrict infiltration, leading to surface accumulation and runoff transport, whereas acidic, organic-rich soils exhibit the highest UDMH sorption capacity.

Du et al. [67] provide strong empirical confirmation of the outcomes presented in this study’s stochastic modeling. Both approaches show a consistent mechanism of UDMH in soils, with moderate mineral surface sorption, diffusion-limited kinetics, and partial nonequilibrium mass exchange. The experimental trends align with the model parameters, confirming that the stochastic framework accurately captures the key physical and chemical processes of UDMH retention and release. It extends lab-observed short-term kinetic patterns to real-world scales, showing weak sorption and slow diffusion lead to prolonged persistence and gradual desorption in heterogeneous soils. These results affirm the model’s robustness and usefulness for predicting the long-term environmental fate of UDMH TPs.

The review shows that reaction terms, including decay and chain-reaction kinetics, were negligible under the calibration conditions. This suggests that the persistence of the solute and TPs is primarily controlled by physical nonequilibrium and reversible sorption processes rather than measurable degradation within the experimental timeframe. This aligns with previous studies showing sorption–desorption hysteresis and domain exchange dominate long-term dynamics of low-volatility contaminants in soils with minimal organic matter [29,31]. Therefore, conducted simulations suggest that UDMH residue persistence results from limited degradation and mass transfer constraints between mobile and immobile water domains, consistent with laboratory degradation studies and field investigations at rocket impact sites, where UDMH and major TPs exhibit half-lives of days to months, depending on environmental conditions [1,27,30].

Another issue related to the affected sites is the varied impacts of the landscape. Empirical studies have shown that surface contamination footprints from major rocket accidents can cover thousands of square meters and include hotspots with UDMH concentrations far exceeding health-based thresholds [8,19,64]. These findings highlight technical challenges for sustainable land management, as cleanup standards and monitoring protocols remain unspecified, thereby granting authorities discretion and flexibility in licensing conditions. Several remediation strategies include controlled burning of UDMH spills [68], adsorption [69], and advanced oxidation processes, such as the Fenton process [70,71], cavitation [72], catalytic wet peroxide oxidation [73,74], and others. All methods leave residual pollutants due to incomplete decomposition or detoxification, requiring excavation, ploughing, and site restoration. Post-restoration monitoring often detects UDMH TPs in soils and vegetation [75], raising concerns about their long-term persistence and environmental impact, which needs further research.

Under SDG 15, special focus is required on restoring degraded land and soil, with the aim of reaching a world where land degradation is neutral [76]. This highlights the importance of preventive site characterization through the use of appropriate analytical models. For instance, the NASA Spill Prevention, Control, and Countermeasures Plan considers pre-launch studies of soil properties, such as assessments of buffering capacity and the soil’s ability to mitigate rocket propellant impacts [77]. Comparatively, current Kazakhstani legislation mandates only a post-incident investigation of contaminated areas, with recommendations formulated for subsequent launches [78]. Moreover, the list of priority pollutants should be expanded to consider the complex impact of crashed vehicles on the local ecosystems.

## 5. Conclusions

This study provides an integrated assessment of the long-term behavior of unsymmetrical dimethylhydrazine transformation products in soils from the rocket crash area near the Baikonur Cosmodrome. The combined application of chemical analysis using Vac-SPME-GC-MS and stochastic modeling through HYDRUS-1D provides new insights into the persistence of UDMH TPs under local environmental conditions. Among the five target compounds, pyrazine (PAN) and 1-methyl-1H-pyrazole (MPA) were consistently detected in soils 15 years after the incident. Their concentrations ranged from 0.04 to 2.35 ng g^−1^ for PAN and 0.06 to 3.48 ng g^−1^ for MPA, with the highest loadings at a depth of 50 cm, decreasing progressively with depth. Both compounds exhibited positive correlations with soil acidity and moisture, indicating that physicochemical soil properties substantially influence the retention and migration of TP.

The stochastic modeling and sensitivity analyses demonstrate that the persistence of UDMH transformation products in soils is primarily governed by physical nonequilibrium transport processes rather than intrinsic degradation kinetics. Even under weak-to-moderate sorption, long-term tailing may sustain quasi-stationary concentrations for hundreds to thousands of days, particularly in soils with higher immobile fractions. These findings suggest that effective remediation should focus on addressing immobile domain storage and slow-release processes, rather than relying solely on natural attenuation.

The results highlight the importance of integrated long-term monitoring and predictive modeling for land management affected by space launch activities, with a focus on the persistence and behavior of UDMH transformation products in soil. Relying solely on surface measurements or short-term degradation data in conventional assessments may underestimate contamination that is sustained by diffusion-limited exchange and reversible sorption processes in subsurface zones. In this context, combining physicochemical soil characterization with stochastic and reactive transport simulations offers a strong framework for identifying potential hotspots, forecasting contaminant redistribution, and supporting evidence-based environmental surveillance. This integrated method enhances the accuracy of risk assessments and facilitates the development of adaptive, science-based policies for managing and restoring areas affected by space launch operations.

## Figures and Tables

**Table 1:** Grouping soils based on their physical–chemical features for calibration.

Group Number	Depth, cm	Representative Soil Sample	pH (Average ± SD)	Moisture Content, % (Average ± SD)	Organic Content, % (Average ± SD)
50-1	50	SE-0–50	8.5 ± 0.8	6.2 ± 0.7	5.4 ± 0.8
50-2		E-5–50	8.1 ± 0.5	8.5 ± 0.7	5.4 ± 0.9
50-3		E-10–50	7.7 ± 0.5	11.5 ± 0.9	5.7 ± 0.4
100-1	100	E-20–100	7.8 ± 0.6	3.2 ± 0.6	3.8 ± 1.0
100-2		C-100	7.7 ± 0.5	8.0 ± 2.8	4.3 ± 0.8
150-1	150	E-5–150	7.5 ± 0.4	2.9 ± 0.7	3.9 ± 1.5
150-2		N-20–150	7.6 ± 0.7	6.5 ± 1.9	5.0 ± 1.8

**Table 2:** Analytical performance of the Vac-HS-SPME-GC-MS method.

Soil Group	Analyte	Concentration Range, ng g^−1^	*R* ^2^	Slope	RSD of Slope, %	LOD, ng g^−1^	LOQ, ng g^−1^
SE 0-50 ^a^	PAN	2.6–100	0.997	42,086	2	0.271	0.904
	MPA	2.6–100	0.996	69,664	3	0.157	0.524
	NDMA	13–500	0.998	3369	2	15.4	51.4
	MTA	52–2000	0.985	910	6	92	306
	PAL	13–500	0.983	4794	6	21	69
SE 0-50 ^b^	PAN	2.6–100	0.958	24,543	9	0.243	0.809
	MPA	2.6–100	0.975	75,606	7	0.133	0.445
	NDMA	13–500	0.961	2540	9	14.7	49.2
	MTA	52–2000	0.988	560	5	48	160
	PAL	13–500	0.973	2464	7	6	20
SE 0-50 ^c^	PAN	2.6–100	0.995	31,094	3	0.142	0.474
	MPA	2.6–100	0.996	96,745	3	0.050	0.167
	NDMA	13–500	0.993	3009	4	9.9	32.9
	MTA	52–2000	0.997	831	2	63	211
	PAL	13–500	0.997	3663	2	14	47
NW 5-50	PAN	2.6–100	0.997	68,257	3	0.137	0.456
	MPA	2.6–100	0.999	127,518	2	0.069	0.231
	NDMA	13–500	0.999	4768	2	0.6	1.9
	MTA	52–2000	0.994	2131	4	11	38
	PAL	13–500	0.996	8186	3	5	17
E 20-100	PAN	2.6–100	0.997	97,694	3	0.218	0.727
	MPA	2.6–100	0.989	257,415	5	0.157	0.523
	NDMA	13–500	0.988	15,633	5	0.29	0.97
	MTA	52–2000	0.993	2644	4	1.5	5.1
	PAL	13–500	0.999	33,334	1	0.3	1.1
N 100	PAN	2.6–100	0.992	773,397	4	0.021	0.071
	MPA	2.6–100	0.996	189,4151	3	0.037	0.123
	NDMA	13–500	0.996	113,073	3	1.43	4.75
	MTA	52–2000	0.990	28,318	4	12.2	40.7
	PAL	13–500	0.997	313,132	3	4	13
E 5-150	PAN	2.6–100	0.990	191,994	5	0.112	0.372
	MPA	2.6–100	0.995	418,065	3	0.060	0.201
	NDMA	13–500	0.991	17,368	4	11.2	37.3
	MTA	52–2000	0.985	7885	6	39	130
	PAL	13–500	0.807	52,204	22	3.6	11.9
N 20-150	PAN	2.6–100	0.998	745,654	2	0.041	0.137
	MPA	2.6–100	0.989	805,802	5	0.061	0.203
	NDMA	13–500	0.995	49,550	3	10.7	35.7
	MTA	52–2000	0.997	21,832	2	33	108
	PAL	13–500	0.992	106,536	4	2	6

^a^ 1st calibration; ^b^ 2nd calibration; ^c^ 3rd calibration.

**Table 3:** MS detection program for UDMH TPs identification in SIM mode.

Analyte	CAS Number	Retention Time, min	Group Start Time, min	Ions, *m/z*	
				Quantification	Confirmation
PAN	290-37-9	2.60	2.0	80	53
MPA	930-36-9	3.03		82	81
NDMA	62-75-9	3.94	3.8	74	42
MTA	6086-21-1	6.91	6.0	83	56
PAL	288-13-1	8.27	7.7	68	41

**Table 4:** Descriptive statistics of PAN and MPA concentrations in soil samples.

UDMH TP	Depth, cm	Samples Above LOD	Concentration, ng g^−1^		SD, ng/g	Skewness Factor	Kurtosis Factor	Concentration, ng g^−1^	
			Mean	Median				Min	Max
PAN	50	29	0.71	0.60	0.40	2.6	9.3	0.21	2.35
	100	22	0.35	0.25	0.28	1.5	2.1	0.04	1.15
	150	18	0.21	0.17	0.16	1.1	0.7	0.04	0.60
MPA	50	31	0.84	0.59	0.69	2.3	6.2	0.13	3.48
	100	1	0.06	0.06	N/A	N/A	N/A	0.06	0.06
	150	21	0.21	0.11	0.26	3.3	11.7	0.07	1.23

## Data Availability

The data used in this study are available upon reasonable request.

## Supplementary Materials

The following supporting information can be downloaded at: https://www.mdpi.com/article/10.3390/toxics13110963/s1. Table S1. UDMH TPs’ maximum permissible concentration in soils according to Kazakhstani legislation. Table S2. Physical and chemical properties of soils. Table S3. Concentrations of standard solutions employed for spiking the selected soil matrices. Figure S1. Physical-chemical properties and concentrations of PAN and MPA in soil samples.
