# Supplementary material for: Hidden Contamination Patterns: A Stochastic Approach to Assessing Unsymmetrical Dimethylhydrazine Transformation Products in Kazakhstan’s Rocket Crash Area
Source: Toxics. 2025 Nov 6;13(11):963. doi: 10.3390/toxics13110963 (PMC12656182; doi:10.3390/toxics13110963)
Supplement: Supplementary file 1 [file toxics-13-00963-s001.zip › toxics-3957499-supplementary.pdf]

toxics

Supplementary Materials

Hidden Contamination Patterns: A Stochastic Approach to Assessing Unsymmetrical Dimethylhydrazine Transformation Products in Kazakhstan's Rocket Crash Area

Ivan Radelyuk [1,*], Aray Zhakupbekova [2,3], Alua Zhumadildinova [2,3], Artem Kashtanov [1,2,3] and Nassiba Baimatova [1,2,3,*]

[1] Department of Chemistry and Chemical Technologies, Toraighyrov University, Pavlodar, Kazakhstan
[2] Faculty of Chemistry and Chemical Technology, Center of Physical Chemical Methods of Research and Analysis, Al-Farabi Kazakh National University, Almaty, Kazakhstan
[3] Environmental and Analytical Chemistry Laboratory, Al-Farabi Kazakh National University, Almaty, Kazakhstan
* Correspondence: radelyuk.i@tou.edu.kz (I.R.); baimatova@cfhma.kz (N.B.)

Table S1. UDMH TPs' maximum permissible concentration in soils according to Kazakhstani legislation.

Analytes	Maximum permissible concentration in soil (mg/kg)
UDMH	0.1
NDMA	0.01
MTA	10.0

Table S2. Physical and chemical properties of soils.

Sample number		Moisture content, %	Organic matter, %	pH
1.	N/50	5.79	7.0	8.2
2.	NE/50	12.9	5.0	7.0
3.	E/50	9.78	4.5	8.3
4.	SE/50	4.76	4.5	9.5
5.	S/50	6.03	4.4	8.6
6.	W/50	7.79	6.5	8.5
7.	NW/50	8.13	5.2	7.8
8.	N-5/50	6.19	5.5	7.8
9.	NE-5/50	7.19	4.9	9.4
10.	E-5/50	8.27	4.0	7.5
11.	S-5/50	5.34	4.1	7.9
12.	SW-5/50	6.10	5.7	8.0
13.	NW-5/50	8.36	6.7	7.7
14.	N-10/50	8.37	5.5	8.0
15.	NE-10/50	6.87	5.7	10.1
16.	E-10/50	11.7	5.6	8.0

 https://doi.org/10.3390/xxxxx

17.	W-10/50	10.7	6.1	8.3
18.	NW-10/50	7.29	6.1	8.4
19.	NE-15/50	9.31	4.2	7.3
20.	E-15/50	7.58	5.1	7.8
21.	NW-15/50	6.21	5.1	8.7
22.	N-20/50	9.12	5.6	7.6
23.	NE-20/50	7.15	5.1	9.3
24.	E-20/50	9.13	5.1	8.6
25.	W-20/50	9.02	6.5	8.9
26.	NW-20/50	11.3	5.8	7.5
27.	N-25/50	5.77	5.9	7.9
28.	NE-25/50	10.9	6.1	7.8
29.	E-25/50	6.58	5.7	7.8
30.	W-25/50	6.22	6.2	7.9
31.	NW-25/50	8.97	5.8	8.5
32.	MU-C/50	7.67	4.6	8.1
33.	N/100	3.45	5.2	7.3
34.	NE/100	3.06	4.7	7.1
35.	E/100	4.49	1.3	7.8
36.	SE/100	2.14	3.1	8.2
37.	S/100	6.73	2.7	7.6
38.	W/100	6.08	4.2	8.0
39.	NW/100	3.15	4.5	7.1
40.	N-5/100	3.40	4.4	7.5
41.	NE-5/100	2.46	2.8	8.7
42.	E-5/100	2.80	4.0	7.8
43.	S-5/100	3.74	4.9	7.7
44.	SW-5/100	2.49	4.3	7.8
45.	NW-5/100	3.70	4.2	8.0
46.	N-10/100	5.52	4.9	8.4
47.	NE-10/100	2.55	3.1	8.4
48.	E-10/100	3.46	4.4	7.8
49.	W-10/100	7.55	5.1	6.8
50.	NW-10/100	7.75	4.8	8.2
51.	NE-15/100	2.93	2.7	7.2
52.	E-15/100	2.73	3.0	7.9
53.	NW-15/100	3.39	4.3	8.2
54.	N-20/100	3.69	4.6	8.1
55.	NE-20/100	4.19	4.8	7.5
56.	E-20/100	2.87	2.9	9.4
57.	W-20/100	8.43	4.1	7.5
58.	NW-20/100	13.7	5.2	7.2

59.	N-25/100	7.14	3.4	8.1
60.	NE-25/100	4.91	4.6	8.0
61.	E-25/100	2.84	3.7	7.4
62.	W-25/100	2.33	2.7	7.4
63.	NW-25/100	4.23	3.9	7.3
64.	MU-C/100	11.7	4.5	7.3
65.	N/150	11.8	8.2	8.2
66.	NE/150	4.45	4.6	7.0
67.	E/150	1.61	3.4	7.3
68.	SE/150	2.29	2.1	7.5
69.	S/150	2.20	4.0	7.5
70.	W/150	3.87	4.7	7.8
71.	NW/150	6.92	4.0	6.9
72.	N-5/150	3.83	8.2	7.1
73.	NE-5/150	6.59	7.1	7.9
74.	E-5/150	3.12	3.4	7.3
75.	S-5/150	4.90	3.0	9.6
76.	SW-5/150	7.39	4.8	7.7
77.	NW-5/150	5.85	5.1	7.7
78.	N-10/150	2.53	3.5	8.1
79.	NE-10/150	4.78	4.2	7.8
80.	E-10/150	6.05	4.4	7.4
81.	W-10/150	6.88	4.0	7.5
82.	NW-10/150	5.89	3.2	7.7
83.	NE-15/150	3.83	8.2	7.1
84.	E-15/150	3.54	2.9	7.3
85.	NW-15/150	3.57	4.9	8.2
86.	N-20/150	6.49	2.4	7.2
87.	NE-20/150	3.84	5.3	7.5
88.	E-20/150	2.73	3.2	8.0
89.	W-20/150	3.14	3.7	7.7
90.	NW-20/150	8.11	7.1	7.2
91.	N-25/150	2.71	5.0	7.2
92.	NE-25/150	3.18	3.9	7.2
93.	E-25/150	7.53	5.3	7.2
94.	W-25/150	2.88	3.2	6.9
95.	NW-25/150	2.69	4.0	7.7
96.	MU-C/150	1.41	1.8	7.5

Table S3. Concentrations of standard solutions employed for spiking the selected soil matrices.

Analyte	Calibration range, ng/g	Concentration, ng/μL					
		Level 1	Level 2	Level 3	Level 4	Level 5	Level 6
PAN	2.6 – 100.0	0.2600	0.5200	1.000	2.500	5.000	10.00
MPA	2.6 – 100.0	0.2600	0.5200	1.000	2.50	5.000	10.00
NDMA	13 – 500	1.300	2.600	5.000	12.500	26.00	50.00
MTA	13 – 500	1.300	2.600	5.000	12.00	26.00	50.00
PAL	52 - 2000	5.200	10.40	20.00	50.00	100.0	200.0

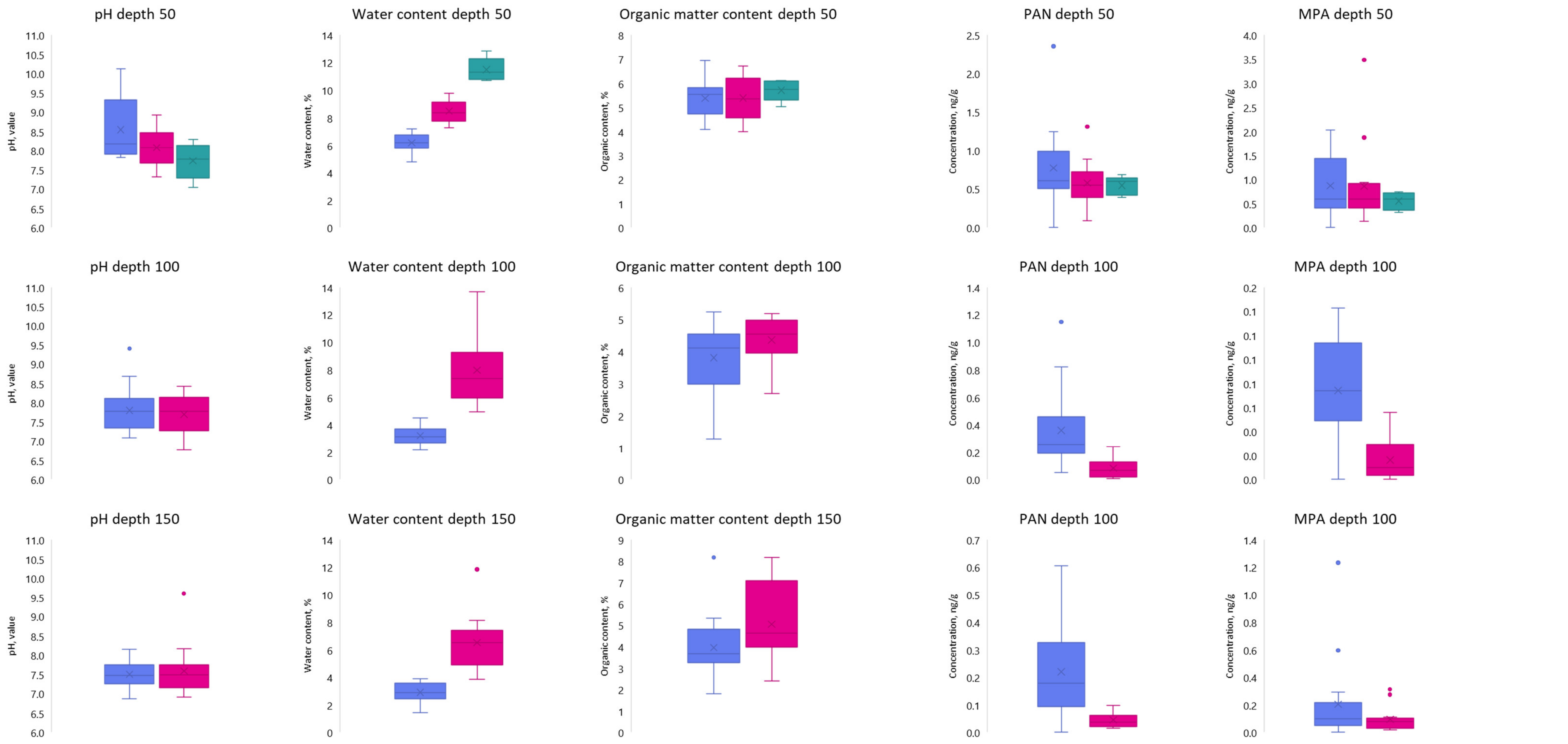

Figure S1. Physical-chemical properties and concentrations of PAN and MPA in soil samples.
